# Effects of dopamine on response properties of ON-OFF RGCs in encoding stimulus durations

**DOI:** 10.3389/fncir.2014.00072

**Published:** 2014-06-30

**Authors:** Lei Xiao, Pu-Ming Zhang, Hai-Qing Gong, Pei-Ji Liang

**Affiliations:** Department of Biomedical Engineering, School of Biomedical Engineering, Shanghai Jiao Tong UniversityShanghai, China

**Keywords:** retinal ganglion cell, dopamine, response latency, firing rate, information coding

## Abstract

Single retinal ganglion cell's (RGCs) response properties, such as spike count and response latency, are known to encode some features of visual stimuli. On the other hand, neuronal response can be modulated by dopamine (DA), an important endogenous neuromodulator in the retina. In the present study, we investigated the effects of DA on the spike count and the response latency of bullfrog ON-OFF RGCs during exposure to different stimulus durations. We found that neuronal spike count and response latency were both changed with stimulus durations, and exogenous DA (10 μM) obviously attenuated the stimulus-duration-dependent response latency change. Information analysis showed that the information about light ON duration was mainly carried by the OFF response and *vice versa*, and the stimulation information was carried by both spike count and response latency. However, during DA application, the information carried by the response latency was greatly decreased, which suggests that dopaminergic pathway is involved in modulating the role of response latency in encoding the information about stimulus durations.

## Introduction

Neuronal response activities contain many aspects, including firing rate, response latency, correlated activity pattern among neurons, etc. How neurons transmit external information via these characteristics is still not fully understood (Averbeck and Lee, 2004; Field and Chichilnisky, 2007). Neuronal firing rate can vary when in exposure to different stimuli, and thus encodes stimulus information (Richmond et al., 1987; Risner et al., 2010). On the other hand, some studies also revealed that the timing of individual spikes, especially the timing of the first spike after stimulus onset (identified as response latency), also played important roles in encoding the information about certain stimulus features, such as stimulus contrast, location, moving speed and direction, etc. (Gawne et al., 1996; Panzeri et al., 2001; Reich et al., 2001; Thiel et al., 2007; Gollisch and Meister, 2008; Risner et al., 2010; Nowak et al., 2011).

In the retina, dopamine (DA) is synthesized and released by dopaminergic interplexiform cells and amacrine cells in the inner retina during exposure to constant or flickering light (Witkovsky, 2004). Studies have shown that DA takes part in regulating circadian rhythmicity, retinal light and dark adaptation process, and contrast sensitivity, etc. (Witkovsky, 2004; Popova and Kupenova, 2011; Jackson et al., 2012). Besides, DA can also modulate neuronal properties, including electrical coupling between retinal neurons and glutamate-gated ionic currents, etc. (Witkovsky and Dearry, 1991; Maguire and Werblin, 1994; Bloomfield and Volgyi, 2009), which results in changes in the response characteristics of retinal ganglion cells (RGCs), such as firing rate, response latency, and receptive field size, etc. (Bonaventure et al., 1980; Witkovsky, 2004; Li et al., 2012).

Visual stimulation contains many important features, such as stimulus intensity, contrast, and duration. Previous study on retinal ERG showed that in the retinal DA-depleted mouse model, amplitudes of retinal ERG a-waves and b-waves (which respectively represented the function of rod photoreceptors and ON bipolar cells) exhibited significant deficits in light-adapted responses and contrast sensitivity (Jackson et al., 2012). In the retina, it was reported that depolarization degree of cone-driven OFF bipolar cells at light offset could be increased with the preceding light ON duration (Schwartz, 1974). And in our previous study, it was also observed that RGCs' responsiveness (including response latency and firing rate) changed with stimulus duration (Xiao et al., 2014). In the present study, we intended to study the effects of DA on the stimulus-duration-dependent response changes and information coding.

Using the multi-electrode recording system, the coding strategy of single bullfrog ON-OFF RGC in response to different stimulus durations, as well as the DA effects on RGC's response and coding ability, was investigated. It was observed that both response latency and spike count of ON response varied with light OFF intervals and *vice versa*. Information analysis showed that response latency and spike count both carried the information about stimulus durations. Application of exogenous DA (10 μM) increased neuronal firing rate and shortened neuronal response latency, and it also attenuated the stimulus-duration-dependent response latency change, and significantly decreased the information carried by the response latency. These results suggest that dopaminergic pathway is involved in modulating the role of response latency in encoding the information about stimulus durations.

## Materials and methods

### Retinal recording

Experiments were performed on isolated bullfrog retinas at room temperature (22–26°C) (Jing et al., 2010; Xiao et al., 2013, 2014). Bullfrogs were dark adapted for about 30 min prior to experiments. A piece of retina (about 4 × 4 mm^2^) was placed on micro-electrode arrays (MEA, MMEP-4, CNNS UNT, USA) with the ganglion cell side contacting the electrodes, and superfused with the oxygenated Ringer's solution. In pharmacological experiment, DA (10 μM) (purchased from Sigma-Aldrich, St. Louis, MO, USA) was applied with the Ringer's solution.

Neuronal activities were recorded by the MEA consisted of 64 electrodes (8 μm in diameter) which were arranged in an 8 × 8 matrix with 150 μm tip-to-tip distance. Signals were amplified by a 64-channel amplifier (MEA workstation, Plexon Inc. Texas, USA; single-end amplifier, amplification 1000×, bandpass 100–8000 Hz), with each channel being sampled at a rate of 40 kHz (along with the stimulus). Spikes from individual neurons were sorted based on principal component analysis (PCA) method (Zhang et al., 2004) as well as the spike-sorting unit in the commercial software Offline Sorter (Plexon Inc. Texas, USA). In order to get accurate data for spike train analysis, only single-neuron events clarified by all the above-mentioned spike-sorting methods were used for further analyses (Li et al., 2012).

All procedures strictly conformed to the humane treatment and use of animals as prescribed by the Association for Research in Vision and Ophthalmology, and were approved by the Ethic Committee, School of Biomedical Engineering, Shanghai Jiao Tong University.

### Stimulation protocols

Light stimuli were projected from a computer monitor onto the isolated retina via a lens system. Before application of stimulation protocols, full-field sustained dim white light (38.9 nW/cm^2^) was given for 30 s to adjust the RGCs' sensitivity to similar levels (Jing et al., 2010; Xiao et al., 2013).

**Figure 1:**
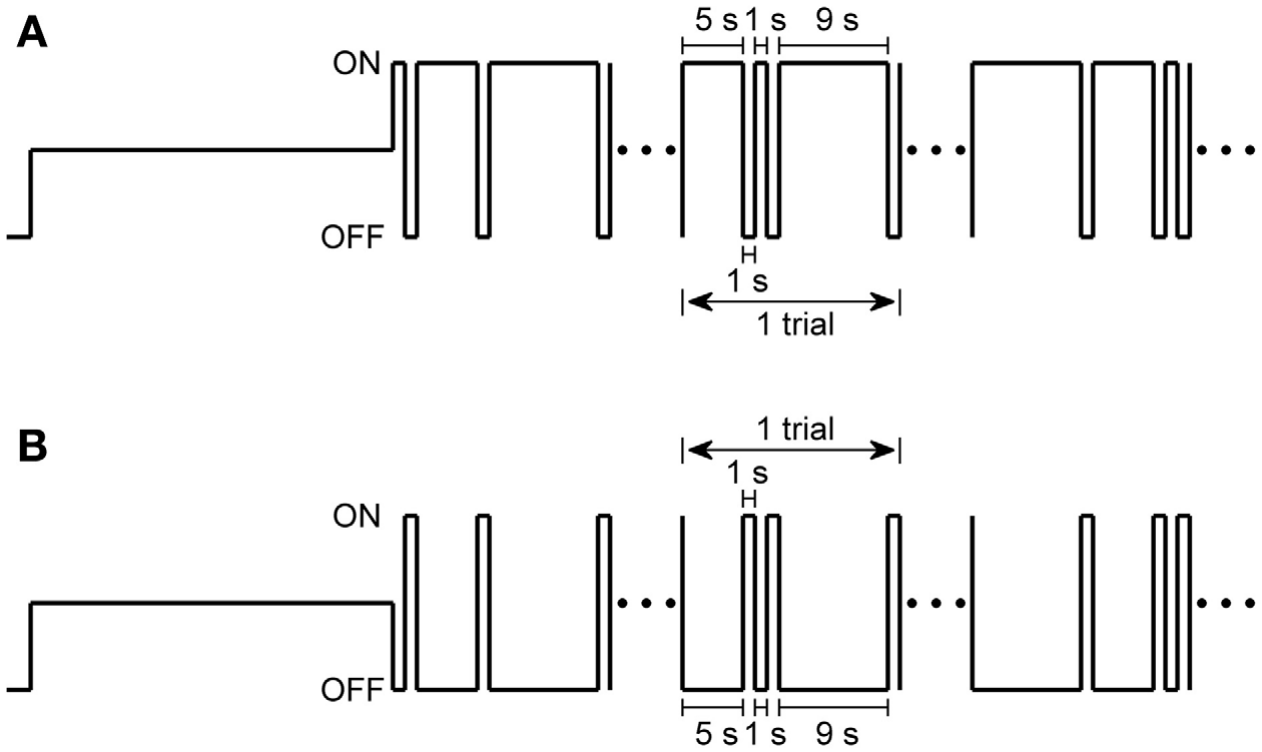
**Two stimulation protocols used in the present study. (A)** Stimulus protocol with different light ON durations, in which 1-s, 5-s, and 9-s light ON were given randomly and separated by 1-s light OFF intervals in each trial, and repeated for 30 trials. **(B)** Stimulus protocol with different light OFF intervals, in which 1-s, 5-s, and 9-s light OFF intervals were presented randomly in each trial and were separated by 1-s light ON. Full-field sustained dim white light was given for 30 s before each stimulation protocol.

In our experiments, two stimulation protocols were applied: (1) the stimulation with different light ON durations, in which light ON stimuli (77.7 nW/cm^2^) with duration of 1, 5, and 9 s were presented randomly in each trial and separated by 1-s full-filed light OFF intervals (about 0.0015 nW/cm^2^), and repeated for 30 trials (Figure 1A). (2) the stimulation with different light OFF intervals, in which randomized light OFF intervals of 1, 5, and 9 s were separated by full-filed 1-s light ON stimuli, and also repeated for 30 trials (Figure 1B).

### Information estimation

In the present study, metric-space method was used to estimate the stimuli information carried by both neuronal spike count and response latency of the first spike after stimulus onset (Victor and Purpura, 1996). The metric-space method measures the distance between two spike trains by three elementary manipulations: adding and deleting spikes at a cost of unity, as well as shifting spike timing, which costs *q* per unit time of moving. The value of *q* expresses the relative sensitivity to the precise timing of the spikes (Victor, 2005).

For a neuron with *N*_*tot*_ spike trains elicited by *N*_*sti*_ different stimuli, the shortest distance (*D*[*q*](*r*_*i*_, *r*_*j*_)) between one spike train (*r*_*i*_) and other spike train (*r*_*j*_) of this neuron (*i*, *j* = 1, 2, …, *N*_*tot*_. and *i* ≠ *j*) is computed based on the above mentioned three elementary manipulations. If one spike train (*r*) is elicited by a stimulus in class *s*_α_, the average distance (*d*(*r*, *s*_γ_)) from the spike train (*r*) to each of the spike trains elicited by stimuli of class *s*_γ_(γ = 1, 2, …, *N*_*sti*_) is defined as (Victor and Purpura, 1996):

(1) $$d(r,\;s_{\gamma })={\left[{\langle D[q]{\left(r,\;r^{\prime }\right)}^{z}\rangle }_{r^{\prime }\text{ elicited by }s_{\gamma }}\right]}^{1/z},$$

where angle brackets denote the average over all the spike trains (*r*′) elicited by a stimulus in class *s*_γ_, and *z* is arbitrarily set as −2 (Victor and Purpura, 1996). After computing the average distance for every spike train, *N*_*tot*_ spike trains can be classified into *N*_*sti*_ response classes. This classification can be summarized by a matrix *N*(*s*_α_, *r*_β_), whose entries indicate the number of times that spike trains elicited by the stimulus class *s*_α_ are classified into response class *r*_β_. If a spike train *r* is elicited by stimulus class *s*_α_(α = 1, 2, …, *N*_*sti*_), it is classified into the response class *r*_β_(β = 1, 2, …, *N*_*sti*_) when *d*(*r*, *s*_β_) is the minimum of all the average distances, and increment *N*(*s*_α_, *r*_β_) by 1. If there are *k* average distances sharing the minimum, elements of the matrix *N* corresponding to these *k* average distances are incremented by 1/*k*.

*N*(*s*_α_, *r*_β_) denotes the number of response sequences elicited by stimulus class *s*_α_ which are classified to be the response elicited by stimulus class *s*_β_. Clustering performance can be quantified by the transmitted information *H* (Victor and Purpura, 1996):

(2) $$\begin{array}{l} H=\frac{1}{N_{tot}}\sum_{\alpha ,\beta }N(s_{\alpha },r_{\beta })[\log_{2}N(s_{\alpha },\;r_{\beta })-\log_{2}\sum_{\alpha }N(s_{\alpha },\;r_{\beta })\\ \;-\log_{2}\sum_{\beta }N(s_{\alpha },r_{\beta })+\log_{2}N_{tot}], \end{array}$$

In our present study, there are three stimulation classes with equal probability (*N*_*sti*_ = 3), the maximal value of transmitted information (*H*) is log_2_3 bits when perfect clustering occurs (*N*(*s*_α_, *r*_β_) = *N*_*tot*_/3 for α = β and others are 0), while random clustering leads to *H* = 0.

*H* value changes with the cost parameter *q*, and we can obtain the information carried by different response component for different *q* value (Victor and Purpura, 1996). When *q* = 0 s^−1^, *H*_0_ represents the amount of information contained in the spike count or firing rate. If the peak value of *H* (*H*_*peak*_) occurs at *q* > 0 s^−1^, it implies that there is some information contained in the temporal structure of spike train. The information contributed by response latency of the first spike is obtained by selecting the first spike in each trial only, and those trials in which no spike fired are excluded (Reich et al., 2001).

### Bias in estimating the information

Estimating the information using Equation 2 with a limited number of trials will cause a sampling bias (Panzeri and Treves, 1996). To estimate this bias, we used Equation 2 to recalculate the information *H* after randomly associating spike trains with stimuli. The average value of 10 such calculations (*H*_*bias*_) is the estimated bias in *H* value estimation (Victor and Purpura, 1997).

## Results

Our experiments were performed on bullfrog retinas. Bullfrog RGCs can be classified into four subtypes based on their response properties: sustained edge detector, convexity edge detector, changing contrast detector, and dimming detector (Maturana et al., 1960; Ishikane et al., 2005). In our present study, more than 90% RGCs recorded were changing contrast detector, they respond transiently to both light ON and OFF stimuli, and hereafter our analyses were focused on such ON-OFF RGCs.

### DA effects on neuronal response latency and spike count of ON-OFF RGCs during exposure to different stimulus durations

In the retina, DA is an important neuromodulator. Activation of DA receptors can influence RGCs' responses (Witkovsky, 2004). In the present study, exogenous DA (10 μM) was applied to study whether DA took part in modulating ON and OFF response characteristics, including firing rate and response latency, during exposure to different stimulus durations.

**Figure 2:**
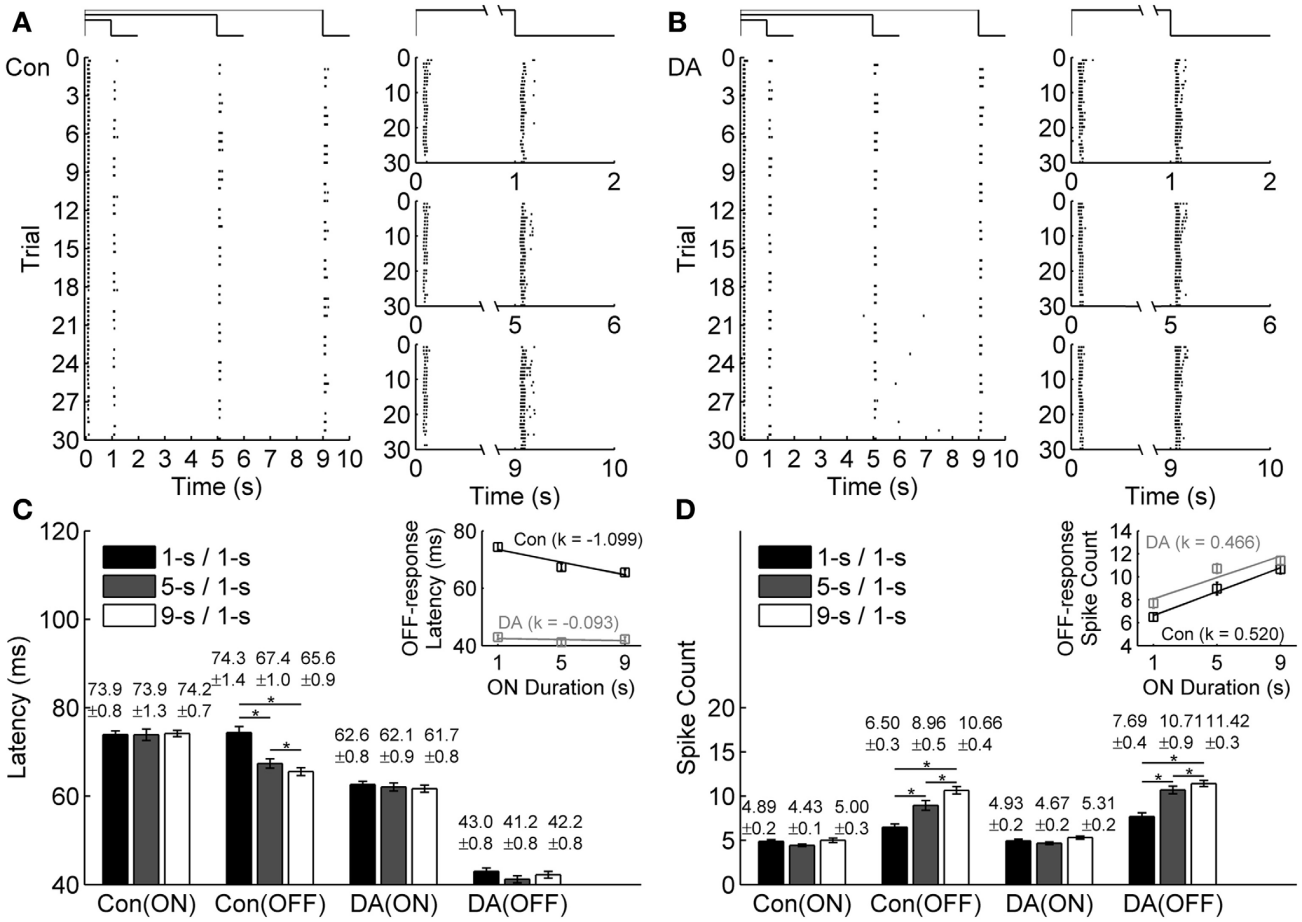
**Effects of DA on neuronal ON and OFF response latencies and spike counts during exposure to different light ON durations. (A,B)** A typical cell's response to different light ON durations in control (Con) and DA conditions, respectively. The left panels show the cell's firing activities in all the trials, the occurrence of each spike is represented by a dot. The right panels show the cell's responses during 1-s/1-s, 5-s/1-s, and 9-s/1-s (ON/OFF) stimulus patterns, respectively. **(C,D)** Statistical results of response latencies and spike counts of the example cell in the control and DA conditions. Insets exhibit linear fitting (*y* = k^*^x + *b*) results about the relationship between latency/spike count of OFF response and light ON duration in the control and DA conditions. *n* = 30 trials. Error bars indicate ± s.e.m., ^*^p < 0.05, paired *t*-test.

Raster plots of an example neuron during exposure to different light ON durations in the control condition and during DA application are plotted in Figures 2A,B, respectively. The timing of the first spike after stimulation switch was defined as the response latency (Greschner et al., 2006; Gollisch and Meister, 2008). Because bullfrog ON-OFF RGCs mostly only fired in the first 200 ms of light ON and OFF transients, only the first 200-ms responses during light ON and OFF stimulations were taken for further analyses in our present study.

Average response latencies and spike counts of the ON and OFF responses of the example neuron during exposure to different light ON durations are plotted in Figures 2C,D. In the control condition, the OFF response latency tended to be shortened and the spike count tended to be increased when light ON duration was prolonged, but the ON response did not exhibit obvious change. During DA application, average response latencies of both ON and OFF responses of this example neuron were shortened and spike counts were increased. On average, it was found that during DA application, ON and OFF response latencies did not exhibit obvious change with light ON duration, but the spike count of OFF response still tended to be increased.

The ON-time-dependent latency change of OFF response was quantified by the slope of linear fitting. For the example neuron, such a change was obviously attenuated during DA application [the linear fitting slope *k* = −1.099 and −0.093 in the control and DA conditions, respectively, while the relative difference of the fitting slopes (|*k*_Con_ − *k*_DA_|/|*k*_Con_ + *k*_DA_|) is 0.8440; Figure 2C inset]. The ON-time-dependent spike count change of OFF response in DA condition was similar to that in the control condition (the linear fitting slope *k* = 0.520 and 0.466 in the control and DA conditions, respectively, while the relative difference of the fitting slopes is 0.0548; Figure 2D inset).

Statistical results obtained from 45 RGCs of 6 retinas show that the OFF response latency was significantly decreased when light ON duration was increased in the control condition (Table 1; paired *t*-test, *p* < 0.05), but in DA condition there was no obvious difference for the OFF response latency during exposure to different light ON durations (Table 1; paired *t*-test, *p* > 0.05). Spike count of OFF response was obviously increased with light ON duration, and such tendency was kept during DA application (Table 1).

**Table 1:** **Average response latency and spike count in response to different light ON durations in the control and DA conditions (Mean ± s.e.m., *n* = 45 RGCs from 6 retinas)**.

**Stimulation (ON/OFF)**			**1-s/1-s**	**5-s/1-s**	**9-s/1-s**
Response latency (ms)	ON-response	Control	78.3 ± 1.4	79.2 ± 1.6	79.8 ± 1.6
		DA	66.2 ± 1.7	67.7 ± 2.9	66.7 ± 2.3
	OFF-response	**Control**	**81.5 ± 4.6**	**73.2 ± 2.4**	**70.3 ± 2.2**
		DA	48.6 ± 3.3	46.3 ± 2.9	47.5 ± 2.9
Spike count	ON-response	Control	3.36 ± 0.42	3.17 ± 0.43	3.29 ± 0.42
		DA	4.49 ± 0.19	4.12 ± 0.175	4.31 ± 0.21
	OFF-response	**Control**	**3.96 ± 0.42**	**7.51 ± 0.65**	**9.56 ± 0.91**
		**DA**	**6.34 ± 0.76**	**10.00 ± 0.89**	**11.62 ± 1.21**

*Bold entries indicate response properties (response latency and spike count) which were significantly changed with light ON durations. p < 0.05, paired t-test*.

It is well acknowledged that light increment and decrement can activate retinal ON and OFF pathways, respectively. Different synaptic circuitries and neurotransmitter receptors of ON and OFF pathways make light response of ON and OFF RGCs show some differences in response sensitivity, temporal kinetics, and receptive field size etc. (DeVries, 1999; Chichilnisky and Kalmar, 2002; Zaghloul et al., 2003; Margolis and Detwiler, 2007). Thus, the effects of DA on response characteristics of ON-OFF RGCs during exposure to different light OFF intervals were further studied.

**Figure 3:**
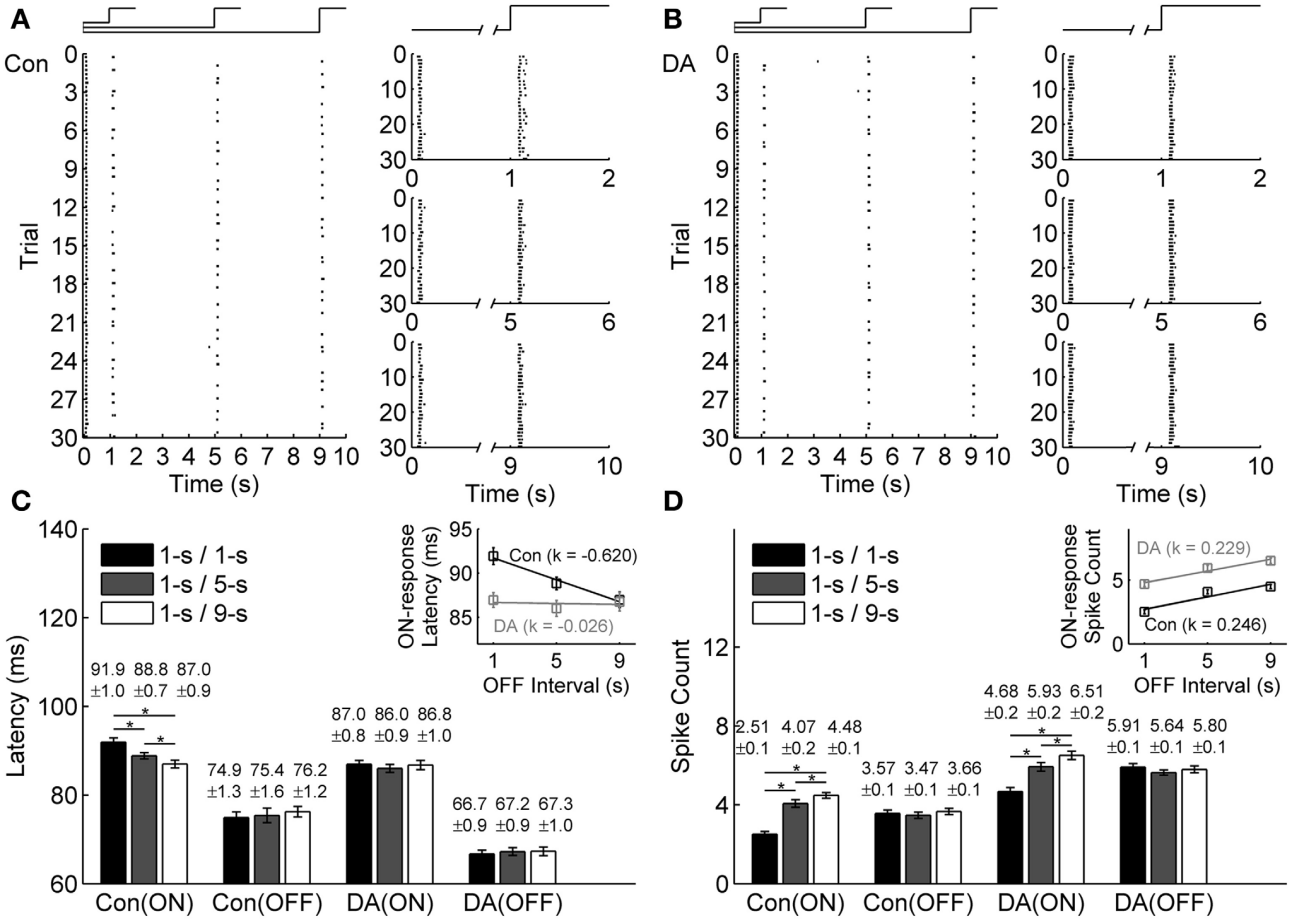
**Effects of DA on neuronal ON and OFF response latencies and spike counts during exposure to different light OFF intervals. (A,B)** A typical cell's response to different light OFF intervals in the control (Con) and DA conditions, respectively. The left panels show the cell's firing activities in all the trials, the occurrence of each spike is represented by a dot; The right panels show the cell's responses during 1-s/1-s, 1-s/5-s, and 1-s/9-s (ON/OFF) stimulus patterns, respectively. **(C,D)** Statistical results of the response latencies and spike counts of the example cell in the control and DA conditions, respectively. Insets exhibit linear fitting results about the response latency and the spike count of ON response with light OFF interval in the control and DA conditions. *n* = 30 trials. Error bars indicate ± s.e.m., ^*^p < 0.05, paired *t*-test.

In exposure to different light OFF intervals, the raster plots of an example neuron in the control and DA conditions are shown in Figures 3A,B, respectively. For this example neuron, only ON response properties were changed, with latency shortened and spike count increased with light OFF interval, while OFF response properties did not exhibit obvious change (Figures 3C,D), which showed that ON-OFF RGCs' activities were mainly modulated by the preceding stimulus. During DA application, it was also observed that DA shortened neuronal response latency and increased neuronal spike count, and the OFF-time-dependent latency change of ON response was obviously attenuated (Figures 3C,D).

The OFF-time-dependent response latency change of ON response was also quantified by the slope of linear fitting, and it was attenuated obviously during DA application (the linear fitting slope *k* = −0.620 and −0.026 in the control and DA conditions, respectively, and the relative difference of the fitting slopes is 0.9195; Figure 3C inset). In DA condition, the spike count of ON response was increased with light OFF interval, but the OFF-time-dependent spike count change of ON response was similar to that in the control condition (the linear fitting slope *k* = 0.246 and 0.229 in the control and DA conditions, respectively, and the relative difference of the fitting slopes is 0.0385; Figure 3D inset).

Statistical results from 23 neurons of 3 retinas also showed that the ON response latency was significantly decreased with light OFF interval in control condition, but in DA condition, it exhibited no obvious difference in exposure to different light OFF intervals, and the OFF-time-dependent spike count change of ON response was still kept with DA application (Table 2).

**Table 2:** **Average response latency and spike count in response to different light OFF intervals in the control and DA conditions (Mean ± s.e.m., *n* = 23 RGCs from 3 retinas)**.

**Stimulation (ON/OFF)**			**1-s/1-s**	**1-s/5-s**	**1-s/9-s**
Response latency (ms)	ON-response	**Control**	**104.0 ± 3.1**	**100.5 ± 2.9**	**98.6 ± 2.9**
		DA	90.5 ± 2.9	90.4 ± 2.8	90.3 ± 2.6
	OFF-response	Control	84.6 ± 3.2	85.5 ± 3.4	84.5 ± 3.5
		DA	75.2 ± 3.7	75.1 ± 4.2	75.7 ± 4.4
Spike count	ON-response	**Control**	**2.84 ± 0.39**	**3.87 ± 0.42**	**4.30 ± 0.43**
		**DA**	**5.04 ± 0.20**	**5.97 ± 0.72**	**6.55 ± 0.91**
	OFF-response	Control	3.69 ± 0.52	3.69 ± 0.51	3.63 ± 0.50
		DA	4.31 ± 0.28	4.11 ± 0.23	4.10 ± 0.21

*Bold entries indicate response properties (response latency and spike count) which were significantly changed with light OFF intervals. p < 0.05, paired t-test*.

### Roles of neuronal response latency and spike count in encoding stimulus durations

Though neuronal response latency and spike count both varied with stimulus durations, many reports suggested that the contribution of neuronal response latency and spike count during information encoding is not equal (Panzeri et al., 2001; Gollisch and Meister, 2008). We then analyzed the contribution of the response latency and the spike count in encoding the information about stimulus durations based on the metric-space method (Equations 1 and 2) (Victor and Purpura, 1996).

**Figure 4:**
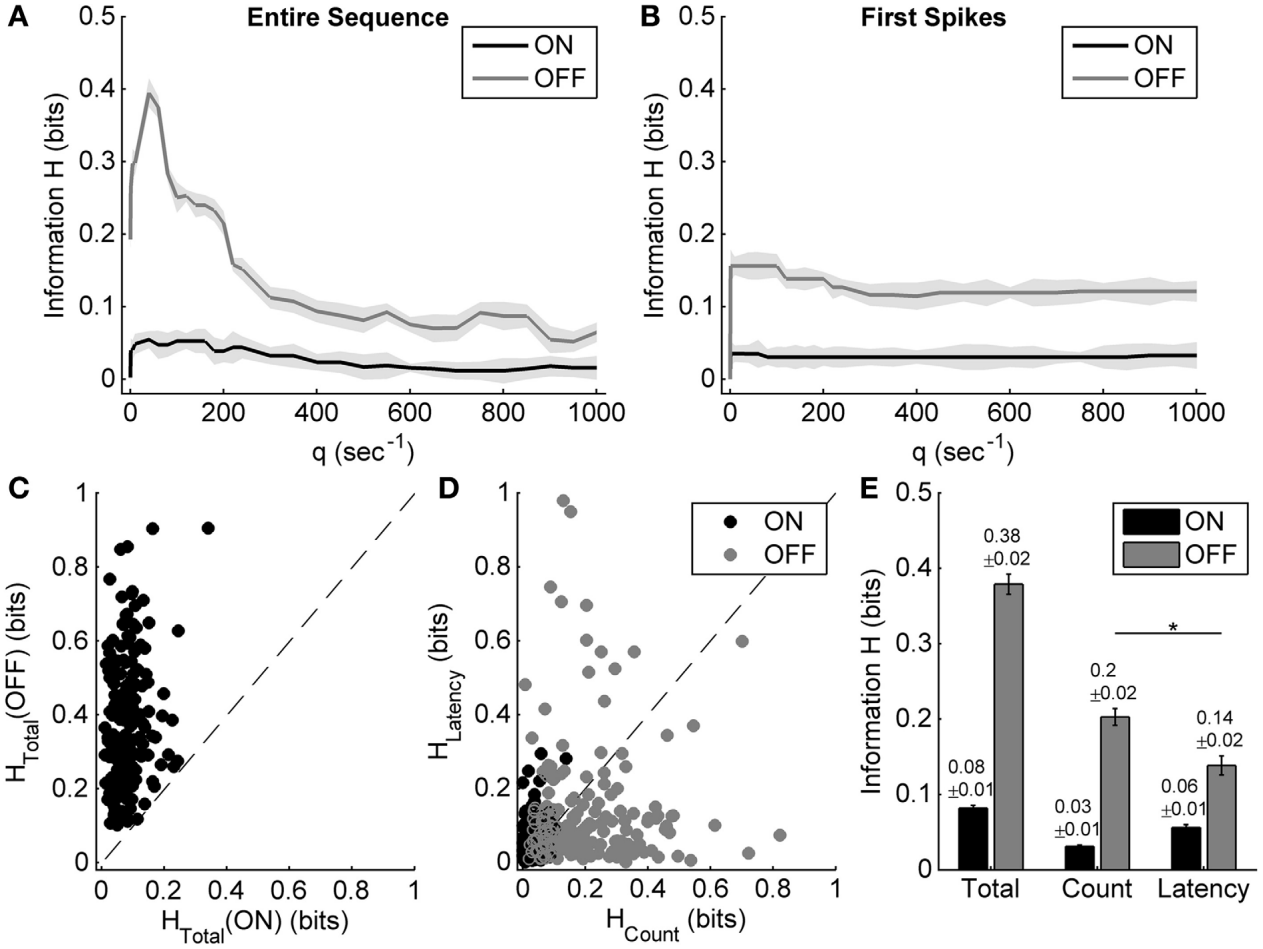
**Information carried by the spike count and the response latency of ON and OFF responses during exposure to different light ON durations. (A,B)** Examples of applying the metric-space method to the entire sequence, and only first spikes of one example neuron's ON and OFF responses during exposure to different light ON durations. Shadows represent the information bias due to limited number of trials. **(C)** Scatter plot of the total information carried by neuronal ON and OFF responses of 179 cells from 10 retinas. **(D)** Scatter plot of information carried by the spike count and the response latency of ON and OFF responses for each neuron. **(E)** Statistical results of the total information, as well as the information carried by the spike count and the response latency. *n* = 179 neurons. Error bars indicate ± s.e.m., ^*^p < 0.05, paired *t*-test.

Figures 4A,B show the results of applying the metric-space method to the entire sequence and only the first spike of one example neuron's ON and OFF responses during exposure to different light ON durations. When considering the entire ON and OFF response sequences (Figure 4A), the total information carried by ON and OFF responses (the maximum information value) and the information carried by the spike count (the information value at the cost *q* = 0 s^−1^) can be estimated, while the information contributed by response latency reaches its maximum value when only the first spike is considered (Figure 4B) (Reich et al., 2001).

For the example neuron, the total information carried by the OFF response was about 0.39 bits, which was obviously higher than that carried by the ON response (0.05 bits); the information carried by the spike count and the response latency of OFF response were 0.19 bits and 0.16 bits, respectively (Figures 4A,B). The OFF response carried more information about light ON duration than the ON response did, which was consistent with the results that OFF response characteristics were changed obviously with light ON duration.

Statistical results from 179 neurons of 10 retinas showed that the total information carried by the OFF response was significantly higher than that carried by the ON response (Figures 4C,E; paired *t*-test, *p* < 0.05). It is also shown that for the OFF response, the information carried by spike count was a little higher than that carried by response latency (Figures 4D,E; paired *t*-test, *p* < 0.05).

**Figure 5:**
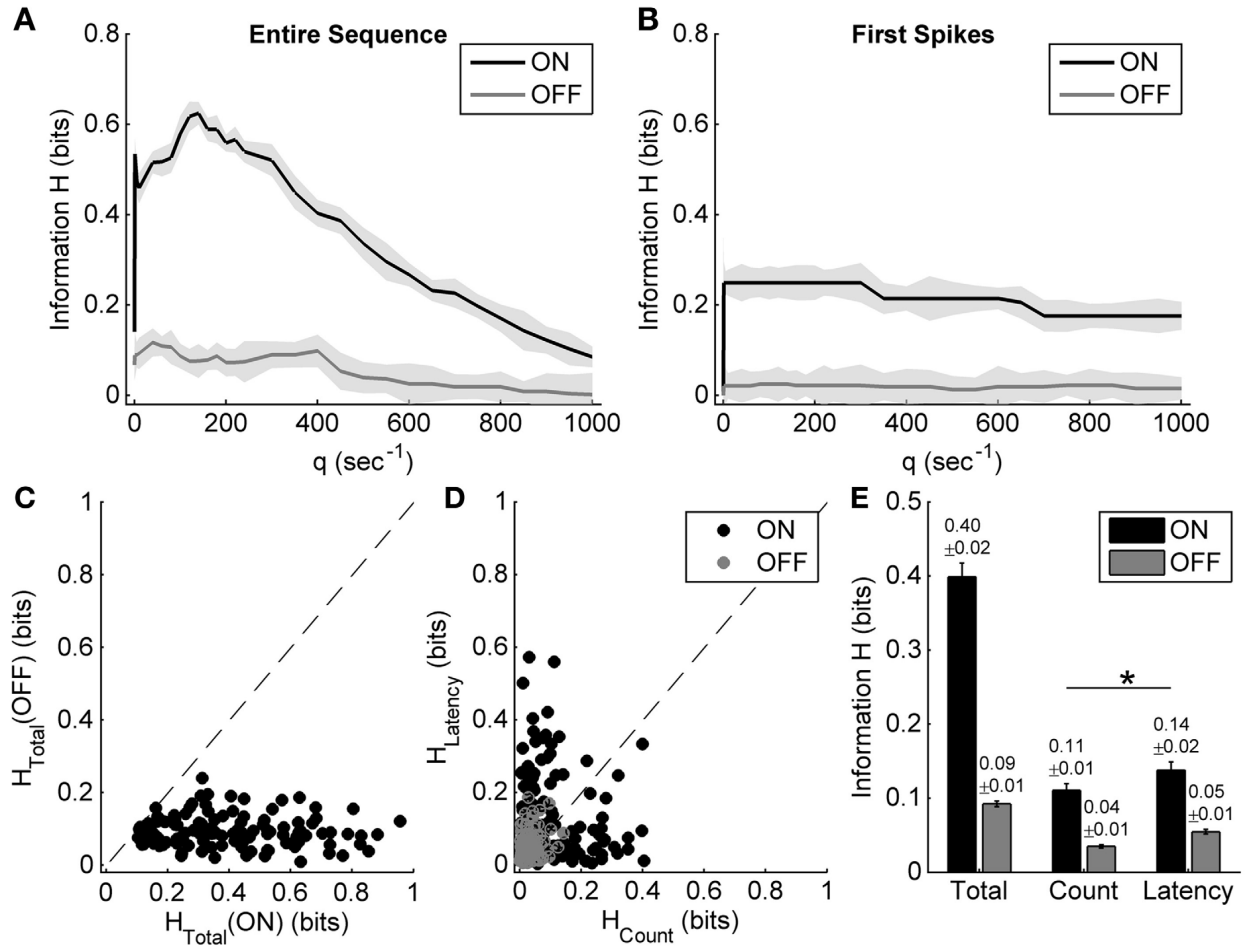
**Information carried by the spike count and the response latency of ON and OFF responses during exposure to different light OFF intervals. (A,B)** Examples of applying the metric-space method to the entire sequence and only first spike of the same neuron's ON and OFF responses during exposure to different light OFF intervals. Shadows represent the information bias due to limited number of trials. **(C)** Scatter plot of the total information carried by neuronal ON and OFF responses of 125 cells from 8 retinas. **(D)** Scatter plot of the information carried by the spike count and the response latency of ON and OFF responses for each neuron. **(E)** Statistical results of the total information, as well as the information carried by the spike count and the response latency. *n* = 125 neurons from 8 retinas. Error bars indicate ± s.e.m., ^*^p < 0.05, paired *t*-test.

Information encoding during exposure to different light OFF intervals was also analyzed based on the metric-space method. Figures 5A,B show the results of applying the metric-space method to the entire sequence and only the first spikes of one example neuron's ON and OFF responses during exposure to different light OFF intervals. For this example neuron, the total information about light OFF interval carried by the ON response (about 0.62 bits) was obvious more than that carried by the OFF response (about 0.12 bits), and the information carried by the spike count of ON response (about 0.14 bits) was less than that carried by the response latency of ON response (about 0.25 bits) (Figures 5A,B). Statistic results from 125 RGCs of 8 retinas also showed that ON response carried more information than OFF response (Figure 5C), and the information carried by the response latency was significantly more than that carried by the spike count (Figures 5D,E, paired *t*-test, *p* < 0.05).

### Influence of DA on information coding

Our results showed that neuronal spike count and response latency both carried the information about stimulus durations. In pharmacological experiments, it was observed that 10 μM DA could attenuate the stimulus-time-dependent response latency change, but it had little effect on the stimulus-time-dependent spike count change. So, the influence DA exerts on the capacity of information carried by the spike count and the response latency about stimulus durations was further examined.

**Figure 6:**
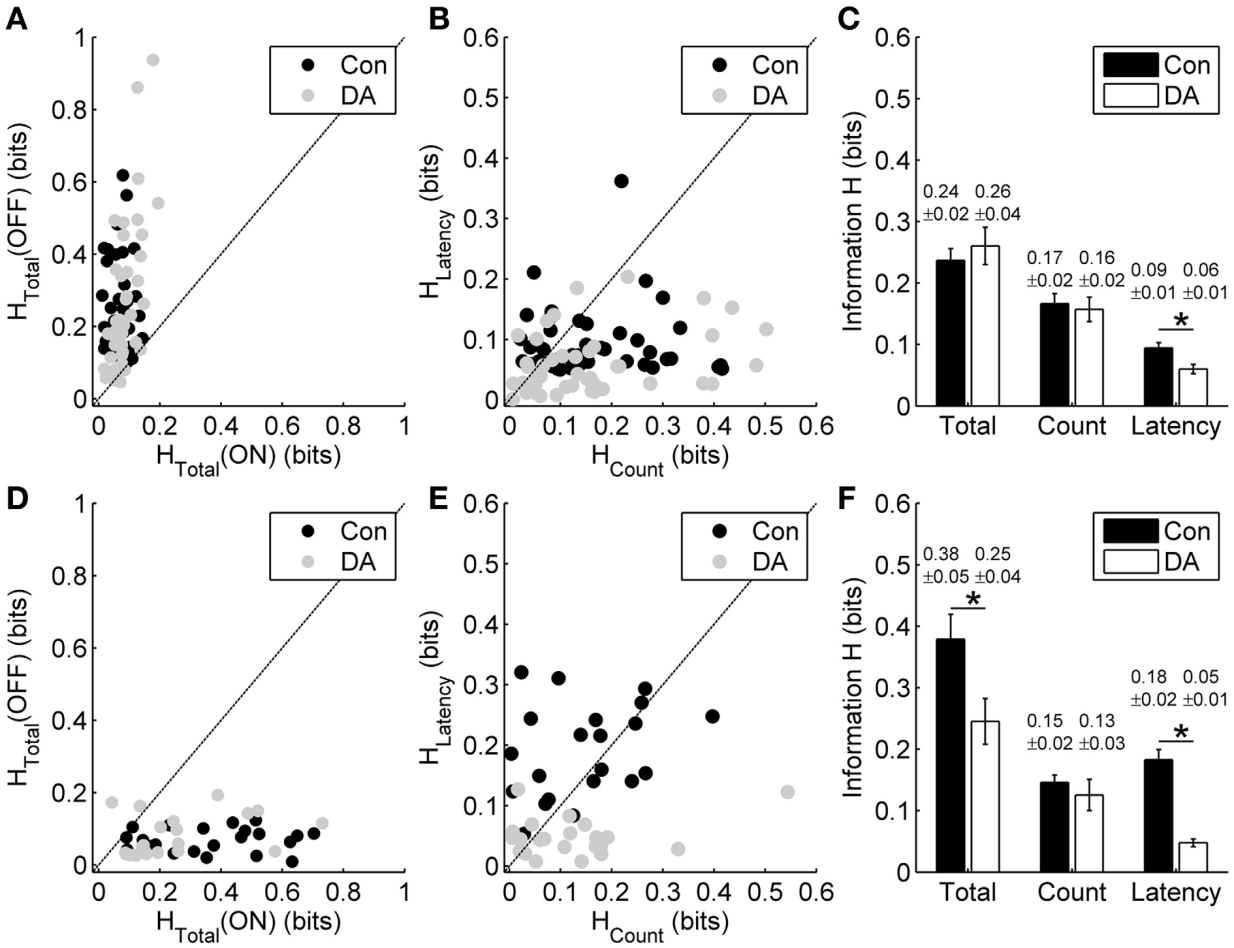
**Effects of DA on spike count coding and response latency coding. (A)** Scatter plot of the total information carried by the entire ON- and OFF-sequence in the control (Con) and DA conditions during exposure to different light ON durations for each neuron. **(B)** Scatter plot of the information carried by the spike count and the response latency of OFF response in the control and DA conditions during exposure to different light ON durations for each neuron. **(C)** Statistical results of the total information carried by the entire OFF-sequence, the information carried by the spike count and the response latency of OFF response in the control and DA conditions during exposure to different light ON durations. *n* = 45 neurons from 6 retinas. **(D)** Scatter plot of the total information carried by the entire ON- and OFF-sequence in the control and DA conditions during exposure to different light OFF intervals for each neuron. **(E)** Scatter plot of the information carried by the spike count and the response latency of ON response in the control and DA conditions during exposure to different light OFF intervals for each neuron. **(F)** Statistical results of the total information carried by entire ON-sequence, the information carried by the spike count and the response latency of ON response in the control and DA conditions during exposure to different light OFF intervals. *n* = 23 neurons from 3 retinas. Error bars indicate ± s.e.m., ^*^p < 0.05, paired *t*-test.

Our results showed that DA did not obviously influence the total information carried by the entire ON- and OFF-sequence in response to different light ON durations (Figure 6A; paired *t*-test, *p* > 0.05, *n* = 45 cells from 6 retinas). On the other hand, in exposure to different light OFF intervals, DA did not obviously influence the total information carried by the entire OFF-sequence (Figure 6D; paired *t*-test, *p* > 0.05, *n* = 23 cells from 3 retinas), but tended to decrease the total information carried by entire ON-sequence (Figures 6D,F; paired *t*-test, *p* < 0.05). Given that RGCs' responses were mainly modulated by the preceding stimuli (Figures 2, 3) and the information about light ON/OFF duration was also mainly carried by OFF/ON response (Figures 4, 5), therefore our experiments were further focused on the effects of DA on information coding of the OFF response during exposure to different light ON durations and *vice versa*.

For the neuronal responses during exposure to different light ON durations, we respectively calculated the information carried by the spike count and the response latency of OFF response in the control and DA conditions based on the metric-space method (Victor and Purpura, 1996). In the control condition, information carried by the spike count of OFF response was little higher than that carried by the response latency. Application of DA significantly decreased the information carried by the response latency (Figures 6B,C; paired *t*-test, *p* < 0.05), but it did not obviously change the information carried by the spike count (paired *t*-test, *p* > 0.05). During exposure to different light OFF intervals, though the response latency of ON response carried more information than the spike count in the control condition, the information carried by the response latency also decreased significantly during DA application (Figures 6E,F; paired *t*-test, *p* < 0.05) and the information carried by the spike count did not changed obviously (paired *t*-test, *p* > 0.05).

Information about stimuli can be carried by neuronal activity only when the response variability is correlated with the stimulation parameters (Borst and Theunissen, 1999). The effects of DA on the information coding by the spike count and response latency were consistent with the effects of DA on the stimulus-time-dependent spike count and response latency changes.

## Discussion

In the present study, the effects of DA on rate coding and latency coding of single bullfrog ON-OFF RGCs in exposure to different stimulus durations were investigated. Spike count and response latency were changed with the stimulus durations, and they both took part in encoding information about stimulus duration. DA at a concentration of 10 μM obviously attenuated the stimulus-duration-dependent response latency change and also decreased the information carried by the response latency. These results suggest that in the retina, dopaminergic pathway is involved in modulating the role of response latency in encoding the information about stimulus durations.

### Effects of DA on the stimulus-duration-dependent response changes

DA is an important endogenous neuromodulator in the retina. It has been reported that RGCs' activities, including response latency and firing rate, can be influenced by DA (Bonaventure et al., 1980; Witkovsky, 2004). In the present study, we observed that DA-related pathway took part in modulating stimulus-duration-dependent response latency changes.

It was reported that in the clawed frog, vitreal DA concentration was measured 564 ± 109 nM in the light-adapted condition, and the retinal DA uptake was saturated when the DA concentration in the bath was about 10 μM (Witkovsky et al., 1993). So, in our present study, 10 μM DA was used to study the DA effects on the stimulus-duration-dependent RGCs' response changes. There are two classes of DA receptors, D1 and D2. Previous studies showed that D2 receptors are more sensitive to DA than D1 receptors, and light-induced dopamine release can desensitize D2 receptors (Witkovsky, 2004), so DA has concentration-dependent effects on different types of receptors. But, in our study, 10 μM DA could saturate the effects of both types of DA receptors, which could help to identify whether DA played a role in modulating the stimulus-duration-dependent responses. Meanwhile, in the present study, we focused on the stimulus-duration-dependent response changes and the effects of DA on it, and our experiments were performed at one level of brightness/contrast. Our experimental results showed that neuronal spike count was increased with stimulus durations at the selected level of brightness/contrast, during control and during 10 μM DA application, which suggested that spike count was not saturated at this concentration of DA and the level brightness/contrast, so it was feasible to study the stimulus-duration-dependent response changes at this concentration of DA and level of brightness/contrast.

DA receptors have been found on retinal neurons, including RGCs (Witkovsky and Dearry, 1991). Activation of D1 and D2 receptors can modulate RGCs' excitability via regulating cAMP-dependent protein kinase in opposite ways (Witkovsky and Dearry, 1991). A study in mouse RGCs showed that blocking D1-type receptors decreased RGCs' response amplitude, whereas blocking D2-type receptors had an opposite effect (Yang et al., 2013). Recent experiments performed on bullfrog retina suggested that light-induced dopamine release activated D1-type receptors and desensitized D2-type receptors (Li et al., 2012), and application of exogenous DA shortened the response latency (Li and Liang, 2013), which was consistent with our present results. So, one possible mechanism for the ON-time-dependent OFF response changes is that prolonged light ON duration can increase DA release (Bloomfield and Volgyi, 2009), which will activate D1-type receptors in ON-OFF RGCs, and eventually increase the OFF responsiveness.

On the other hand, RGCs mainly receive excitatory inputs (glutamate) from bipolar cells, and modulation of bipolar cells' activities can directly influence RGCs' responses, especially the response latency. It was reported that the depolarization degree of cone-driven OFF bipolar cells at light offset could be increased with the preceding light ON duration (Schwartz, 1974), which should result in elevated the OFF responsiveness of RGCs. In the retina, bipolar cells receive glutamatergic input from cones, and DA can enhance glutamate-gated currents (Maguire and Werblin, 1994), which can elevate the depolarization degree of cone-driven OFF bipolar cells at light offset and RGCs' firing activities. DA release from dopaminergic amacrine cell is increased by light ON (Bloomfield and Volgyi, 2009). Another possible mechanism for the ON-time-dependent OFF response changes observed in our experiments is that prolonged light ON duration can increase DA release (Bloomfield and Volgyi, 2009), which will enhance glutamate-gated current in the retina, and eventually shorten the response latency and enhance the firing rate of OFF response.

However, DA release is decreased during darkness, so the DA effect can hardly explain the OFF-time-dependent ON response changes. It was reported that retinal ON and OFF pathways are asymmetric (DeVries, 1999; Chichilnisky and Kalmar, 2002), with the main difference being that ON bipolar cells possess metabotropic glutamate receptors (mGluRs) and OFF bipolar cells possess ionotropic glutamate receptors (iGluRs). Different mechanisms underlying the activation of these two types of receptors make postsynaptic cells exhibit opposite response polarities, activation of mGluRs indirectly closes cause cation channels through a signaling cascade that involves G-protein, while glutamate-gated cation channels are directly opened when glutamate binds to iGluRs (Yang, 2004; Oesch et al., 2011).

For cone-driven ON bipolar cells, it was reported that their activities can be depressed by the intracellular calcium concentration via inhibitory feedback to cation channels (Snellman et al., 2008). So, one possible mechanism for the OFF-time-dependent ON response changes is that prolonged light OFF interval can increase glutamate released by cones (Yang, 2004; Oesch et al., 2011), which results in a decrement in the intracellular calcium concentration in ON bipolar cells and increases these cells' activity to the following light ON stimulation, and eventually elevates the ON responsiveness of RGCs.

In general, the timing of the first spike after stimulation switch depends on the direct excitatory glutamatergic pathway from photoreceptors to ganglion cells via bipolar cells in the retina, but the firing rate depends on both direct excitatory input (glutamate) from bipolar cells and lateral inhibitory modulation (GABA and glycine) from amacrine cells. In the retina, DA can enhance glutamate-gated current (Maguire and Werblin, 1994), our results showed that during 10 μM DA application, the stimulus-duration-dependent response latency change was attenuated, which suggested that DA-application eliminated the stimulus-duration-dependent glutamate-gated current change. However, DA had no significant effect on the stimulus-duration-dependent firing rate change. Though application of exogenous DA (10 μM) caused an increase in RGCs' firing rate, the stimulus-duration-dependent firing rate change may be attributed to other mechanisms, such as the activities of GABAergic and glycinergic networks related to amacrine cells, as well as the stimulus-duration-dependent glutamate release by photoreceptors (Schmitz and Witkovsky, 1996).

### Coding strategies of retinal ON and OFF pathways

Spike count and response latency are basic and important neuronal response properties, which are both involved in neuronal information coding. Some experimental studies showed that response latency could convey information about stimuli in addition to that encoded by spike count (Panzeri et al., 2001; Reich et al., 2001; Chase and Young, 2007; Storchi et al., 2012). Quantitative analysis also showed that for some neurons, stimulus information was more carried by the spike count, but some other neurons might carry more information by the response latency (Panzeri et al., 2001; Reich et al., 2001; Storchi et al., 2012), which is similar to our results (Figures 4, 5). Our statistical results showed that the response latency of ON response carried more information than the spike count when in exposure to different light OFF intervals, but the spike count of OFF response carried more information about light ON durations, which suggested that ON and OFF pathways are asymmetric in encoding stimulus durations.

As mentioned, bipolar cells in retinal ON and OFF pathways exhibit asymmetric properties (Oesch et al., 2011). It was reported that ON cone bipolar cells can cross-inhibit OFF bipolar cells and OFF RGCs through the activation of AII amacrine cells (Margolis and Detwiler, 2007; Oesch et al., 2011), and thus extend the dynamic range of signaling in the OFF pathway (Manookin et al., 2008). Furthermore, ON and OFF RGCs have different excitatory and inhibitory synaptic inputs, which results in different spike time and spike count variability in these cells (Uzzell and Chichilnisky, 2004; Murphy and Rieke, 2006).These differences in the neural network between retinal ON and OFF pathways may induce different encoding strategies in neuronal ON and OFF responses (Zaghloul et al., 2003; Masland, 2012; Harris and Mrsic-Flogel, 2013; Xiao et al., 2013).

In addition, some other temporal patterns of neuronal response, such as inter-spike intervals and the precise timing of spikes other than the first one, may also carry stimulation information (Reich et al., 2001), which is considered as the residual information and it can be estimated by the difference between the total information carried by the entire sequence and that carried by the spike count and the response latency (Reich et al., 2001). In our present study, the residual information carried by the OFF response in exposure to different light ON durations (about 0.04 ± 0.01 bits, Mean ± s.e.m., Figure 4) was obviously less than that carried by the ON response in exposure to different light OFF intervals (0.15 ± 0.02 bits, Mean ± s.e.m., Figure 5). In the metric-space method, the value of *q* expresses the relative sensitivity to the precise timing of the spikes (Victor, 2005). In the present study, the temporal precision limitation for information capacity (Reich et al., 2001), which was a measure of the precision with which spike times can be used to distinguish one stimulus from others, was defined as 1000/*q*_*max*_, where *q*_*max*_ was the value of *q* at which *H*(*q*) was the peak value of information, and it was found that ON response had more precise spike timing (1000/*q*_*max*_ = 12.5 ± 2.6 ms, Mean ± s.e.m.) than OFF response (1000/*q*_*max*_ = 26.6 ± 3.7 ms, Mean ± s.e.m.) in distinguishing stimulus durations. Given that response latency is also one component of the temporal pattern of neuronal response, these results further suggest that ON and OFF responses of bullfrog ON-OFF RGCs may adopt different strategies.

### Effects of DA on information coding

DA plays an important modulatory role in the retina, it can modulate retinal circadian clock, visual sensitivity, and gap-junctional connectivity between neurons, etc. (Li and Dowling, 2000; Witkovsky, 2004). Some studies also showed that DA could modulate the spatial and temporal pattern of RGCs' activities, and these modulations might exert effects on visual information processing (Li et al., 2012; Bu et al., 2014). It was recently reported that application of exogenous DA did not influence the tendency of neuronal firing rate change in exposure to different stimulation patterns, but decreased the correct rate of population-activity-based stimulation pattern discrimination (Li and Liang, 2013). In our present study, exogenous DA (10 μM) was used to probe the effects of DA on RGCs' responsiveness to different stimulus durations, and it was also observed that DA did not influence the stimulus-time-dependent spike count change, but reduced the visual information encoded by the response latency of singe RGCs.

Neurons can carry stimulus information only when the response variability is correlated with the stimulation parameters (Borst and Theunissen, 1999). In our present study, although exogenous DA (10 μM) elevated neurons' responsiveness (Maguire and Werblin, 1994), it only attenuated the stimulus-time-dependent response latency change without affecting the stimulus-time-dependent spike count change. Hence, DA only decreased the information carried by response latency. These results suggested that in the retina, dopaminergic pathway may modulate the role of response latency in encoding the information about stimulus durations.

## Conclusions

In the present study, exogenous DA (10 μM) and one level of brightness/contrast were used to probe the effects of DA on RGCs' responsiveness to different stimulus durations, we observed that DA obviously attenuated the stimulus-duration-dependent response latency change, but had little effect on the stimulus-duration-dependent firing rate change. Information analysis also showed that DA obviously decreased the information carried by the response latency. These results suggest that dopaminergic pathway takes part in modulating the role of response latency in encoding the information about stimulus durations.

## Author contributions

Research questions: Lei Xiao, Pei-Ji Liang. Experimental design: Lei Xiao. Performed experiments: Lei Xiao, Hai-Qing Gong. Data analysis: Lei Xiao, Pu-Ming Zhang, Pei-Ji Liang. Manuscript preparation: Lei Xiao, Pu-Ming Zhang, Pei-Ji Liang.

### Conflict of interest statement

The authors declare that the research was conducted in the absence of any commercial or financial relationships that could be construed as a potential conflict of interest.
